# The Prevalence of Liver Steatosis and Fibrosis Assessed by Vibration-Controlled Transient Elastography and Controlled Attenuation Parameter in Apparently Healthy Romanian Medical Students

**DOI:** 10.3390/diagnostics11122341

**Published:** 2021-12-13

**Authors:** Robert Nastasa, Carol Stanciu, Sebastian Zenovia, Ana-Maria Singeap, Camelia Cojocariu, Catalin Sfarti, Irina Girleanu, Stefan Chiriac, Tudor Cuciureanu, Laura Huiban, Cristina-Maria Muzica, Anca Trifan

**Affiliations:** 1 Department of Gastroenterology, Grigore T. Popa University of Medicine and Pharmacy, 70015 Iasi, Romania; robertnastasa948@gmail.com (R.N.); sebastianzenovia20@gmail.com (S.Z.); anamaria.singeap@yahoo.com (A.-M.S.); cameliacojocariu@yahoo.com (C.C.); cvsfarti@gmail.com (C.S.); gilda_iri25@yahoo.com (I.G.); stefannchiriac@yahoo.com (S.C.); drcuciureanutudor@gmail.com (T.C.); huiban.laura@yahoo.com (L.H.); lungu.christina@yahoo.com (C.-M.M.); ancatrifan@yahoo.com (A.T.); 2 Institute of Gastroenterology and Hepatology, “St. Spiridon” University Hospital, 700111 Iasi, Romania

**Keywords:** vibration-controlled transient elastography, nonalcoholic fatty liver disease, liver fibrosis, obesity, screening

## Abstract

Vibration-Controlled Transient Elastography (VCTE) with Controlled Attenuation Parameter (CAP) is used as a non-invasive method for evaluating liver steatosis and fibrosis simultaneously. In this prospective study, we aimed to assess the prevalence of liver steatosis and fibrosis, as well as the associated risk factors in Romanian medical students by VCTE and CAP score. We used a cut-off CAP score of ≥248 dB/m for the diagnosis of mild steatosis (S1), ≥268 dB/m for moderate steatosis (S2), and ≥280 dB/m to identify severe steatosis (S3). For liver fibrosis, the cut-off values were: ≤5.5 kPa, indicating no fibrosis (F0), 5.6 kPa for mild fibrosis (F1), 7.2 kPa for significant fibrosis (F2), 9.5 kPa for advanced fibrosis (F3), and 12.5 kPa for cirrhosis (F4). In total, 426 Romanian medical students (67.8% females, mean age of 22.22 ± 1.7 years) were evaluated. Among them, 352 (82.6%) had no steatosis (S0), 32 (7.5%) had mild steatosis (S1), 13 (3.1%) had a moderate degree of steatosis (S2), and 29 (6.8%) had severe steatosis (S3). Based on liver stiffness measurements (LSM), 277 (65%) medical students did not have any fibrosis (F0), 136 (31.9%) had mild fibrosis (F1), 10 (2.4%) participants were identified with significant fibrosis (F2), 3 (0.7%) with advanced fibrosis (F3), and none with cirrhosis (F4). In conclusion, the prevalence of liver steatosis and fibrosis is low among Romanian medical students.

## 1. Introduction

Nonalcoholic fatty liver disease (NAFLD) is a significant health issue that has become the most frequent cause of chronic liver disease, affecting one-quarter of adults worldwide [1]. Moreover, NAFLD is also a growing risk factor for hepatocellular carcinoma (HCC) and a leading indication for liver transplantation [2,3]. NAFLD includes a spectrum of conditions, from simple steatosis, which is considered to be the “benign form” to nonalcoholic steatohepatitis (NASH), the ”progressive form” with different histological features, which is associated with the development of liver fibrosis, including cirrhosis and eventually HCC [4,5].

There are several studies regarding the increasing prevalence of NAFLD in young adults, which parallels the high rates of obesity and metabolic syndrome in this age group [6,7]. The term “young adult” is very familiar to oncologists and refers to a population of patients starting from the age of 20 years without being able to set the upper age limit in clinical practice [8]. Among these patients, some present a number of risk factors for developing NAFLD, such as: obesity, type 2 diabetes mellitus (T2DM), unhealthy lifestyle, smoking habits, and male sex. The interplay between these factors on a background of genetic predisposition may contribute to the installation of NASH [7].

Liver biopsy (LB) is still considered the “imperfect” gold standard method for the staging of liver fibrosis [9,10] and for differentiating between NASH and simple steatosis [11]. However, LB is an invasive method, with several drawbacks, such as sampling errors, intra and inter-observer variability, high cost, limited accessibility, poor patient acceptance, and, also, a low concrete risk of morbidity and mortality, all of which rule out its use as a screening method in apparently healthy individuals [11]. Abdominal ultrasonography (US) is a widely used non-invasive imaging test for first-line hepatic steatosis detection but has low sensitivity, and hence is limited only to severe cases (hepatic fat accumulation more than 20–30%) [12,13].

Nowadays, VCTE is considered the optimal non-invasive method for assessing liver fibrosis, recommended by guidelines over the years for fibrosis evaluation, especially in chronic viral hepatitis [14,15]. Shortly, this technique measures the speed of a mechanically generated shear wave using pulse-echo ultrasonic acquisitions in a much larger hepatic parenchyma, its propagation velocity being directly related to hepatic stiffness [16]. This method is fast, easy to perform, unaching, and also has important repeatability and reproducibility, often being used in clinical practices all over the world [17]. In addition, in recent years, the implementation of CAP (that reflects the fat impedance in the liver) in FibroScan^®^ (Echosens, Paris, France) devices has allowed the concomitant evaluation of hepatic fibrosis and steatosis [18,19]. Compared to US, CAP measurements allow us to identify less severe degrees of steatosis due to it being a quantitative technique with important sensitivity and specificity [20].

Herein, we aimed to evaluate the prevalence of steatosis and fibrosis in apparently healthy Romanian medical students by VCTE and CAP score. We also researched risk factors associated with hepatic steatosis and fibrosis in this population group.

## 2. Materials and Methods

### 2.1. Participants

This study population consisted of apparently healthy 3rd and 5th-year medical students, with a high level of education, from “Grigore T. Popa” University of Medicine and Pharmacy Iasi, evaluated between February and June 2021. Demographic data, personal history, clinical examination, and data obtained from their general practitioner were recorded along with anthropometric and FibroScan assessments. Eligibility criteria were the absence of significant alcohol consumption (<20 g/day in women, <30 g/day in men) and of a history of chronic liver disease. Participants with unreliable transient elastography examination (<10 valid measurements with an interquartile range/median (IRQ/M) ratio >30%) were excluded. For subjects with an LSM value ≥ 7.2 kPa on VCTE examination, laboratory data (platelet count, aspartate and alanine aminotransferase (AST, ALT), gamma-glutamyl transpeptidase (GGT), alkaline phosphatase (ALP), total bilirubin, fasting glucose, total cholesterol, triglycerides, low-density lipoprotein cholesterol (LDL-c), HBs Ag, and anti-HCV Ab) were collected. This study was approved by the Ethics Committee of our university and was conducted according to the principles of the Declaration of Helsinki. Each student signed a written informed consent.

### 2.2. LSM and CAP Assessment

All students were evaluated using FibroScan^®^ 502 Touch (Echosens, Paris, France) by one experienced physician with more than 1000 explorations performed before, using one single examination on each subject, following procedure instructions [14,21]. Briefly, the examination started with the M probe on the right hepatic lobe through the 9th to 11th-intercostal spaces on the midaxillary line after overnight fasting. Switching on the XL probe was considered according to machine indications for obese participants. LSM were expressed in kilopascals (kPa), with the following cut-offs for liver fibrosis: ≤5.5 kPa—F0 (without fibrosis) 5.6 kPa—F1 (mild), 7.2 kPa—F2 (significant), 9.5 kPa—F3 (advanced), and 12.5 kPa—F4 (cirrhosis) [22]. Liver steatosis measured by CAP was expressed in decibels/meter (dB/m), and steatosis degrees were S1 (mild)—248 dB/m, S2 (moderate)—268 dB/m, and S3 (severe)—280 dB/m [18].

### 2.3. Anthropometric Measurements

Height and weight measurements were performed using a height meter and the weight scale. Waist circumference was measured with a height meter and recorded as the midpoint of the distance between the lower border of the rib cage and the iliac crest. Body mass index (BMI) and waist-to-height ratio (WtHR) were calculated as surrogate markers of adiposity [23]. Overweight (≥25 kg/m^2^) and obesity (>30 kg/m^2^) were established using cut-off values defined by the World Health Organization [24], while WtHR is defined by dividing waist circumference (cm) to height (cm), with a settled value ≥0.50 [25]. Waist circumference values of ≥80 cm in women and ≥94 cm in men were defined for abdominal obesity [26], also being considered a surrogate marker of visceral adiposity [23].

### 2.4. Statistics

Statistical analyses were performed using SPSS software version 22.0 (IBM SPP Inc., Chicago, IL, USA). Qualitative data were expressed as numbers (percentage), while quantitative variables were expressed as means ± standard deviation (SD). The Kolmogorov–Smirnov test was used for distribution analysis, continuing with the Student’s *t*-test, Mann–Whitney U, or chi-square test that was considered appropriate for comparing group variables. The association between two variables was made by utilizing the Pearson correlation coefficient (r). Two-tailed *p*-values of <0.05 were considered statistically significant.

## 3. Results

### 3.1. Participants Characteristics

**Figure 1:**
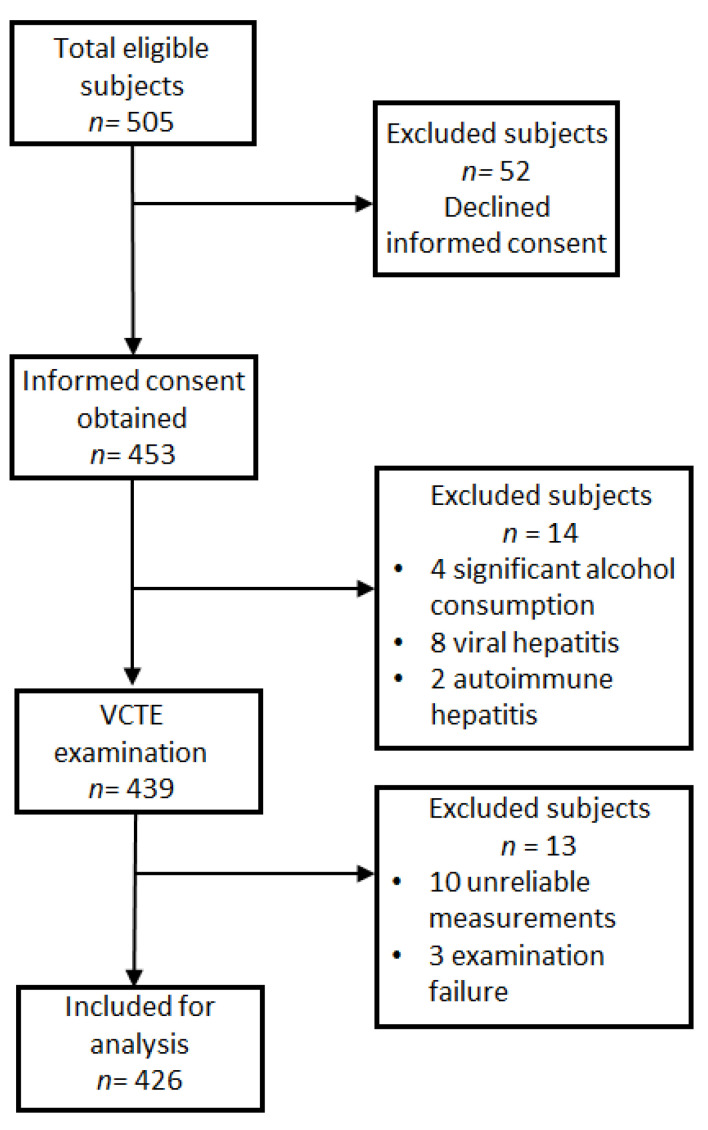
Participant flow-chart.

A total of 505 subjects were invited to participate in this study, 439 of which were evaluated by VCTE and CAP (Figure 1). A total of 13 participants were excluded due to unreliable measurements (10 cases) and examination failure without any measurements (3 students).

Four hundred and twenty-six medical students who met the admission standards were included in the final analysis. All baseline characteristics are summarized in Table 1. The prevalence of overweight, obesity, and abdominal obesity was 14.8%, 3.5%, and 7.5%, respectively. Most of the participants were in the 21-year-old group, with a predominance of female gender (67.8% females, mean age 22.22 ± 1.7 years, and BMI 22.59 ± 3.34 kg/m^2^). Men were heavier (74.09 ± 13.29 kg vs. 61.95 ± 11.56 kg, *p* < 0.001), taller (176 ± 10.2 cm vs. 167 ± 10.7 cm, *p* < 0.001), with a greater proportion of overweight (19.7% vs. 12.5%, *p* = 0.004), obesity (5.8% vs. 2.4 %, *p* = 0.037), and abdominal obesity (12.4% vs 4.5%, *p* < 0.001) than women. Although males had a significantly increased BMI (23.71 ± 3.33 kg/m^2^ vs. 22.07 ± 3.22 kg/m^2^, *p* < 0.001) and WC (78.79 ± 11.35 cm vs. 71.31 ± 8.82 cm, *p* < 0.001), WtHR was not significantly different between groups (0.442 ± 0.06 vs. 0.42 ± 0.05, *p* = 0.159). The prevalence of hepatic steatosis among all students was 17.4%, with a mean CAP of 215.76 ± 48.38 dB/m; 32 (43.2%) of them had S1, and 42 (56.8%) had significant steatosis (S2-S3) with a CAP score above 268 dB/m. The proportion of male students among steatosis degrees was higher compared to women (*p* = 0.026), with an increased CAP value (234.49 ± 47.38 dB/m vs. 206.95 ± 46.42 dB/m, *p* < 0.001). Regarding the prevalence of liver fibrosis, the majority (277 students, 65%) had no liver fibrosis, while 136 (31.9%) participants had F1, 10 (2.4%) had F2, 3 (0.7%) had F3, and no one was found with F4 liver fibrosis, with a mean LSM of 5.29 kPa ± 1.35. Subjects with a liver fibrosis ≥ F2 were predominantly males (61.5%) with a mean BMI of 24.58 ± 3.41 kg/m^2^ and a WtHR 0.462 ± 0.07. Moreover, according to laboratory assessments, four participants had elevated liver enzymes, five had hypercholesterolemia, seven had hypertriglyceridemia, and six of them had increased values of fasting serum glucose, while HBs Ag or anti-HCV Ab were absent in all cases (Table 2).

### 3.2. Participants Characteristics according to Absence or Presence of Liver Steatosis

**Figure 2:**
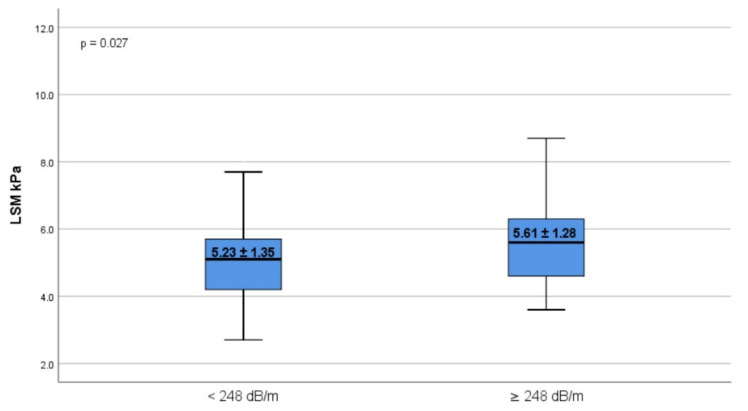
Distribution of LSM values according to absence or presence of liver steatosis. The bottom and the top of each box represent the 25th and 75th percentiles, while the lines through the box indicate the median. The error bars indicate the 10th and 90th percentiles. LSM, liver stiffness measurement.

The participants included in the study that were diagnosed with liver steatosis were predominantly males (*p* = 0.031), with an increased weight (*p* < 0.001), BMI (*p* < 0.001), WC (*p* < 0.001), and WtHR (*p* < 0.001) (Table 3). However, the proportion of overweight (40.5% vs. 9.4%, *p* < 0.001) and obese (9.5% vs. 2.3%, *p* < 0.001) students was significantly higher among the liver steatosis group, of whom 18.9% had abdominal obesity. Regarding liver fibrosis stages, 32 (43.2%) of students had mild fibrosis (F1), 5 (6.8%) had significant fibrosis (F2), and 3 (4.1%) had advanced liver fibrosis (F3), with an increased LSM value (*p* = 0.027) (Figure 2) compared with those without hepatic steatosis, consisting of 104 (29.6%) with F1, 5 (1.4%) with F2, and none with advanced liver fibrosis (*p* = 0.024).

### 3.3. Correlation between Anthropometric Parameters, CAP and LSM

**Figure 3:**
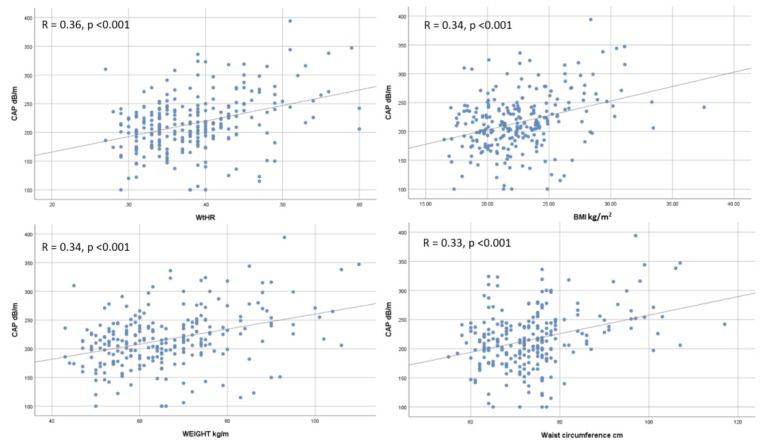
Correlation between CAP and WtHR (**A**), BMI (**B**), Weight (**C**), and Waist circumference (**D**).

**Figure 4:**
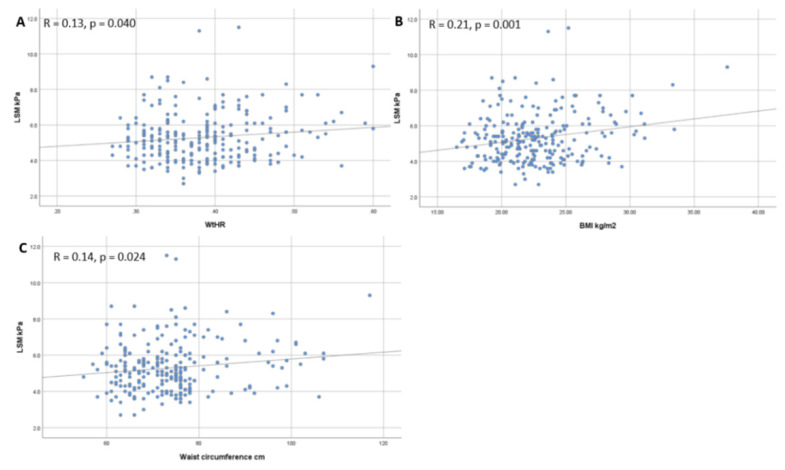
Correlation between LSM and WtHR (**A**), BMI (**B**), and Waist circumference (**C**).

Overall, we found a significant correlation between CAP and WtHR (r = 0.36, *p* < 0.001) (Figure 3A), BMI (r = 0.34, *p* < 0.001) (Figure 3B), weight (r = 0.34, *p* < 0.001) (Figure 3C), and waist circumference (r = 0.33, *p* < 0.001) (Figure 3D). Regarding liver fibrosis expressed by LSM, only WtHR (r = 0.13, *p* = 0.040) (Figure 4A), BMI (r = 0.21, *p* = 0.001) (Figure 4B), and waist circumference (r = 0.14, *p* = 0.024) (Figure 4C) maintained a significant correlation.

## 4. Discussion

Even though NAFLD is a very frequent cause of chronic liver disease, affecting approximately 25% of the world population, it is very clear that NASH and related significant fibrosis are the greatest predictors of high mortality, liver cirrhosis, and HCC [2]. Considering that LB has several shortcomings, multiple non-invasive methods have been developed in the last few years for the evaluation of liver fibrosis and steatosis and are currently used in clinical practice [18,27]. As NAFLD is mostly asymptomatic in the early stages of chronic liver disease, screening techniques are very useful nowadays for risk stratifying in patients with this condition [28]. Magnetic resonance elastography (MRE) is the most accurate non-invasive method for detecting both hepatic steatosis and fibrosis and differentiating patients with advanced fibrosis from those with nonadvanced fibrosis. However, the availability and cost of the MRE represent a major limitation in clinical practice. VCTE may, therefore, offer several advantages, including greater patient acceptability, easiness to perform this technique, and lower costs than MRE [29].

The most important study that analyses the prevalence of NAFLD in young adults was conducted by Mrad et al. in the United States of America, on a population aged from 18 to 35 years, which showed that the prevalence of NAFLD has risen 2.5 times in the last three decades, affecting 25% of the young adults nowadays [6]. Moreover, the authors concluded that the implementation of a screening program is needed in this age group to prevent the development of cirrhosis and its complications.

To the best of our knowledge, this is the first study on the prevalence of NAFLD and liver fibrosis among Romanian medical students. In our study, approximately one in five students, who were apparently healthy, had hepatic steatosis, and one in thirty-three had significant liver fibrosis (≥F2). In addition, in line with the current literature, we have demonstrated that male gender, BMI, waist circumference, and waist-to-height ratio were the main risk factors associated with hepatic fat accumulation.

Most of the students included in our study had no hepatic steatosis (S0: 352; 82.6%) with a CAP score <248 dB/m, and the proportion of patients with mild (S1: 32; 7.5%) and significant steatosis defined by a CAP score ≥268 dB/m (S2–S3: 42; 9.9%) was very low. These results are quite similar to those reported by recent studies. In similar research, Kaya et al. reported a 23.2% NAFLD prevalence in a group of 112 medical students with a mean CAP value of 205.6 ± 43.8 dB/m [30]. Moreover, Abeysekara et al. conducted a study in Great Britain, which included only apparently healthy young adults, and found that the prevalence of hepatic steatosis was 20.7%, with significant steatosis (≥S2) in approximately two-thirds of the patients diagnosed with steatosis based on VCTE (31). Moreover, in our group of participants diagnosed with steatosis, we found that the male sex is more prevalent (*p* = 0.031), with higher BMI (*p* < 0.001), waist circumference (*p* < 0.001), WtHR (*p* < 0.001), and an increased LSM value (*p* = 0.027) than those without steatosis. These risk factors are independently associated with high values of CAP score (mean CAP: 199.16 ± 35.39 dB/m in the group without steatosis vs. 280.41 ± 38.95 dB/m in the steatosis group). Our findings are in accordance with other recent studies, which found that BMI and increased adiposity were the best predictors of hepatic steatosis [7,31].

In our research, the majority of participants had no liver fibrosis (F0: 277; 65%) or had only a mild form (F1: 136; 31.9%), while significant (F2: 10; 2.4%), and advanced fibrosis (F3: 3; 0.7%) were found in a very small proportion of students (3.1%). Additionally, we noticed that LSM maintained a significant correlation with WtHR (r = 0.13, *p* = 0.040), BMI (r = 0.21, *p* = 0.001), and waist circumference (r = 0.14, *p* = 0.024). Our data are in line with those presented by Abeysekara et al., who reported that 2.7% of the participants had LSM values equivalent to METAVIR F2–F4 (42 had significant fibrosis—F2; 45 had advanced fibrosis—F3; 9 had cirrhosis—F4) and, furthermore, the authors reported the same risk factors (BMI, WtHR) for increased LSM values as our study [31]. By contrast, Petta et al., in a general population study that included 890 adults (mean age𢀓53 ± 14 years), found that 3.1% of the participants diagnosed with NAFLD had advanced fibrosis (≥F3) [32]. By comparison, our findings showed that only three students (0.7%) associated both NAFLD and advanced fibrosis, a difference most likely due to the young age of our participants (mean age 22.22 ± 1.7 years). Using the identical non-invasive technique in a similar group of participants (age range 19–22 years) for the evaluation of liver fibrosis and steatosis, Shaheen et al. found that the prevalence of NAFLD among Egyptian young adults was very high (47.5% had variable stages of steatosis) and 56.7% had fibrosis [33]. When compared to our findings, these results seem to be significantly different, and a possible reason could be the demographic contrast between the studied cohorts, considering the increased incidence of obesity and metabolic syndrome (MS) in the Egyptian population. Moreover, in a recent study published in Korea by You et al., which included 159 participants, the authors found that the prevalence of significant fibrosis among apparently healthy subjects was approximately 2.5 times higher (6.9%) than in our research [34]. These contradictory results may be attributed to a high mean age in the Korean cohort (56 ± 10.6 years), increased mean BMI (24.3 ± 3.1 kg/m^2^), demographic contrast, and elevated CAP score values (mean CAP: 248.3 ± 44.4 dB/m). Similar results are reported by two recent studies, which showed a prevalence of significant liver fibrosis between 5.6% and 7.5% in the general population without known chronic liver disease [35,36], suggesting that an increased number of apparently healthy individuals are at high risk of chronic liver disease.

In our group of young adults, the prevalence of overweight and obesity was observed in only 14.8% and 3.5% of the participants, respectively. These findings are in disagreement with other studies conducted in our country. Roman et al. reported in a cohort of patients aged between 18 and 39 years of age a prevalence of overweight and obesity of 22.22% and 9.9%, respectively [37]. On the contrary, other colleagues from our country found obesity in more than half (52.1%) of the participants between 20 and 39 years of age, with a slight difference in favour of women (52.1% vs. 47.3%) [38]. By comparison, our results showed that abdominal obesity was present in only 32 (7.5%) of the students and the highest percentage was found in men (12.4% vs. 4.5%). These discrepancies can be accounted for by the young age of our cohort (mean BMI: 22.22 ± 1.7 kg/m^2^), increased level of medical education and preventive healthcare, and higher socioeconomic status.

Our study had some limitations, however. First of all, we could not perform blood tests to evaluate the levels of triglycerides, high-density lipoprotein cholesterol, and fasting plasma glucose, and therefore, establish the prevalence of MS in our cohort. We decided to exclude laboratory parameters from our research because most of the participants (85% out of 316) refused to undergo venous blood sampling. Moreover, biochemical liver function tests are necessary for minimizing the risk of confounding factors for overestimation of LSM, such as elevated liver enzymes. Secondly, patients diagnosed with NAFLD or significant liver fibrosis (F ≥ 2) based on VCTE and CAP did not perform LB on account of ethical issues that appeared in our population study, which included only apparently healthy participants. Thirdly, our results are based on a single examination for LSM and CAP with M and XL probes without using distinct cut-off values for fibrosis and steatosis assessment. Fourthly, considering the lack of agreement regarding LSM cut-off values for ruling out advanced fibrosis, we used the cut-off values proposed by Wong et al., with the highest AUROC values for significant fibrosis and cirrhosis of 0.84 and 0.95, respectively [22]. Moreover, as the majority of our population had a normal BMI, we used the cut-off values for CAP recommended by current research [18]. Finally, our results may have limited sustainability in different populations and settings as they are derived from a single-centre study. Nevertheless, the prospective design of this study countervails these drawbacks, as it includes a large series of asymptomatic patients with a high level of education. An advanced imaging technique, such as VCTE with CAP, was used for establishing the diagnosis of liver fibrosis and steatosis, methods validated and correlated with histological findings based on LB in NAFLD patients [17].

## 5. Conclusions

In summary, our findings show that the prevalence of steatosis and significant fibrosis among our cohort of apparently healthy medical students is low. In addition, we identified that overweight and obesity were not very common, but high BMI, WtHR, and WC values are associated risk factors for liver steatosis, as well as fibrosis. Therefore, the growing obesity epidemic can be avoided by a multidisciplinary approach to include lifestyle changes with special attention to regular physical exercise. Furthermore, individualized screening strategies should be established for significant liver fibrosis and steatosis according to anthropometric indices.

## Figures and Tables

**Table 1:** The Characteristics of the overall participants included in the study according to gender.

	Overall Cohort *n*, 426	Men *n*, 137	Women *n*, 289	*p*-Value
Age (years)	22.22 ± 1.7	22.45 ± 1.8	22.11 ± 1.6	0.144
Females, *n* (%)	289 (67.8)	-	-	
Weight (kg)	65.84 ± 13.37	74.09 ± 13.29	61.95 ± 11.56	<0.001
Height (cm)	170 ± 8.56	176 ± 10.2	167 ± 10.7	<0.001
Body mass index (kg/m^2^)	22.59 ± 3.34	23.71 ± 3.33	22.07 ± 3.22	<0.001
Waist circumference (cm)	73.7 ± 10.29	78.79 ± 11.35	71.31 ± 8.82	<0.001
Abdominal obesity, *n* (%)	32 (7.5%)	17 (12.4%)	13 (4.5%)	<0.001
Waist-to-height ratio	0.427 ± 0.06	0.442 ± 0.06	0.42 ± 0.05	0.159
Non-overweight, *n* (%)	348 (81.7)	102 (74.5)	246 (85.1)	0.046
Overweight, *n* (%)	63 (14.8)	27 (19.7)	36 (12.5)	0.004
Obese, *n* (%)	15 (3.5)	8 (5.8)	7 (2.4)	0.037
Liver steatosis, *n* (%)	74 (17.4)	39 (28.5)	35 (12.1)	0.011
Steatosis degree, *n* (%)				0.026
0	352 (82.6)	98 (71.5)	254 (87.9)	
1	32 (7.5)	18 (13.1)	14 (4.8)	
2	13 (3.1)	5 (3.7)	8 (2.8)	
3	29 (6.8)	16 (11.7)	13 (4.5)	
Fibrosis stage, *n* (%)				0.186
0	277 (65)	79 (57.6)	198 (68.5)	
1	136 (31.9)	50 (36.5)	86 (29.8)	
2	10 (2.4)	6 (4.4)	4 (1.4)	
3	3 (0.7)	2 (1.5)	1 (0.3)	
CAP, dB/m	215.76 ± 48.38	234.49 ± 47.38	206.95 ± 46.42	<0.001
LSM, kPa	5.29 ± 1.35	5.36 ± 1.2	5.26 ± 1.42	0.582
M-probe, *n* (%)	402 (94.4)	128 (93.4)	274 (94.8)	0.410
XL-probe, *n* (%)	24 (5.6)	9 (6.7)	15 (5.2)	0.372

*n*-number of subjects; CAP, controlled attenuation parameter; LSM, liver stiffness measurement.

**Table 2:** Increased clinical and laboratory parameters in patients with liver fibrosis ≥ F2.

	Subjects, *n* = 13	Increased, *n* (%)
Age (years)	22.7 ± 1.5	-
Males, *n* (%)	8 (61.5)	
Body mass index (kg/m^2^)	24.58 ± 3.41	8 (61.5)
Waist-to-height-ratio	0.462 ± 0.07	5 (38.5)
Platelet count (G/L)	287 ± 72.45	0 (0)
ALT (IU/L)	24.7 ± 14.9	3 (23.1)
AST (IU/L)	26.3 ± 11.4	4 (30.7)
GGT (IU/L)	25.1 ± 16.6	2 (15.3)
ALP (IU/L)	62.7 ± 20.2	0 (0)
Total bilirubin (mg/dL)	0.68 ± 0.25	0 (0)
Fasting glucose (mg/dL)	88.3 ± 17.1	6 (46.1)
Total cholesterol (mg/dL)	208.5 ± 38.3	5 (38.5)
Triglycerides (mg/dL)	131.6 ± 52.9	7 (53.8)
LDL-c (mg/dL)	112.1 ± 26.6	3 (23.1)

ALT, alanine aminotransferase; AST, aspartate aminotransferase; GGT, gamma-glutamyl transferase; ALP, alkaline phosphatase; LDL-c low density lipoprotein cholesterol. Increased values: BMI > 25 kg/m^2^; WtHR > 0.5; ALT > 35 IU/L; AST > 35 IU/L; GGT > 40 IU/L; ALP > 140 IU/L; Total bilirubin > 1 mg/dL; Fasting glucose > 100 mg/dL; Total cholesterol > 200 mg/dL; Triglycerides > 150 mg/dL; LDL-c > 130 mg/dL.

**Table 3:** Baseline characteristics of participants according to the presence of liver steatosis.

	No Hepatic Steatosis *n*, 352	Hepatic Steatosis *n*, 74	*p*-Value
Age (years)	22.18 ± 1.61	22.36 ± 1.73	0.565
Males, *n* (%)	98 (27.8)	39 (52.7)	0.031
Weight (kg)	63.14 ± 11.37	75.09 ± 16.06	<0.001
Height (cm)	170 ± 10.5	171 ± 10.8	0.061
Body mass index (kg/m^2^)	22.14 ± 3.04	24.89 ± 3.91	<0.001
Waist circumference (cm)	71.9 ± 8.82	81.23 ± 12.94	<0.001
Abdominal obesity, *n* (%)	18 (5.1%)	14 (18.9%)	<0.001
Waist-to-height ratio	0.418 ± 0.05	0.482 ± 0.09	<0.001
Non-overweight, *n* (%)	311 (88.3)	37 (50)	0.029
Overweight, *n* (%)	33 (9.4)	30 (40.5)	<0.001
Obese, *n* (%)	8 (2.3)	7 (9.5)	<0.001
Fibrosis stage, *n* (%)			0.024
0	243 (69)	34 (45.9)	
1	104 (29.6)	32 (43.2)	
2	5 (1.4)	5 (6.8)	
≥3	0 (0)	3 (4.1)	
LSM kPa	5.23 ± 1.35	5.61 ± 1.28	0.027
CAP dB/m	199.16 ± 35.39	280.41 ± 38.95	<0.001
M-probe, *n* (%)	341 (96.9)	61 (82.4)	0.244
XL-probe, *n* (%)	11 (3.1)	13 (17.6)	<0.001

CAP, controlled attenuation parameter; LSM, liver stiffness measurement.

## Data Availability

The data presented in this study are available on request from the corresponding author. The data are not publicly available because they are the property of the Institute of Gastroenterology and Hepatology, Iasi, Romania.
